# Genetic Relatedness Promotes Equal Contributions of Males and Females to Brood Care in a Biparental Cichlid Fish

**DOI:** 10.1002/ece3.72570

**Published:** 2025-12-03

**Authors:** Timo Thünken, Janosch Diederich, Simon Vitt, Ranê Aytaç

**Affiliations:** ^1^ Bonn Institute for Organismic Biology, Section II: Animal Biodiversity University of Bonn Bonn Germany

**Keywords:** inclusive fitness, intersexual competition, kin recognition, kin selection, parental cooperation, sexual conflict

## Abstract

Kin discrimination, that is, the differential treatment of kin and nonkin has been studied in a wide range of social contexts because kin selection theory predicts that genetic relatedness among interacting individuals can reduce conflicts and promote cooperation. However, the impact of kin discrimination on parental cooperation in biparental systems is neglected so far. Here, we compared the individual contributions of males and females to brood care of kin and nonkin pairs in *Pelvicachromis taeniatus* , a biparental cichlid fish with a pronounced kin‐mating preference. We conducted a cross‐fostering experiment to control for offspring effects on parental care and an experiment without brood manipulation. In both experiments, the relative contribution of the sexes differed depending on the degree of relatedness within a pair. In unrelated pairs, females cared more than males, whereas the sexes contributed equally in related pairs. Our results indicate that relatedness affects the resolution of sexual conflict. More equal contribution may suggest higher cooperation between sexes within related pairs. Enhanced parental cooperation may be an important benefit of inbreeding, which might have contributed to the evolution of inbreeding preference in *P. taeniatus* , but it may also explain inbreeding tolerance found in other biparental systems.

## Graphical abstract

Here, we compared the individual contributions of males and females to brood care of kin and nonkin pairs in *Pelvicachromis taeniatus* , a biparental cichlid fish with pronounced kin‐mating preference. We found that in unrelated pairs, females cared more than males, whereas the sexes contributed equally in related pairs suggesting higher cooperation between sexes within related pairs. Enhanced parental cooperation may be an important benefit of mating with kin.

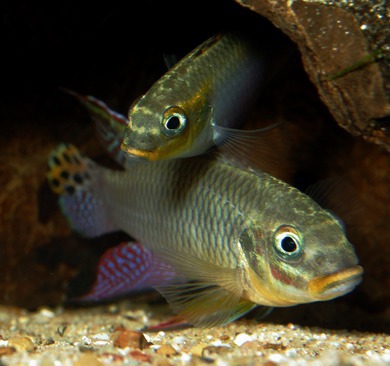

## 1 Introduction

Kin discrimination refers to the differential treatment of kin and nonkin (Mateo 2004; Penn and Frommen 2010) and plays a central role for the evolution of cooperation and social behavior as well as in mate choice (Tregenza and Wedell 2000). Kin selection theory (inclusive fitness theory) predicts that genetic relatedness among interacting individuals can modify the outcome of conflicts and cooperation, that is, increase cooperation and reduce conflicts (Hamilton 1964; West and Gardner 2010). While the importance of kin cooperation is well studied in different nonsexual contexts ranging from alloparental care and sibling competition (Kramer and Meunier 2019) to shoaling (Ward and Hart 2003), the impact of relatedness on parental cooperation between males and females in biparental systems has received little attention. Such neglect may result from the common view that mating with kin, that is, inbreeding, is detrimental for individual fitness because of inbreeding depression, that is, the reduced fitness of inbred offspring (Charlesworth and Willis 2009; Crnokrak and Roff 1999) and, thus, should be generally avoided (Pusey and Wolf 1996). In this view, mating among relatives is considered nonadaptive.

In biparental species, brood care critically depends on the cooperation between parents (Royle et al. 2012). Sexual conflict over brood care is a strong selective force (Houston et al. 2005; Royle et al. 2002), which arises from the differential interest of males and females concerning the tradeoff between investment in the current and future broods (Arnqvist and Rowe 2005; Lessells 2012). As care is costly, each parent aims to save energy for future reproduction and tries to reduce their own contribution at the expense of the partner at the current breeding event. The resolution of the sexual conflict among genetically unrelated individuals has received major attention (Harrison et al. 2009). Resolutions of conflicts include task specialization, negotiation (Smiseth 2019), or manipulation (Paquet and Smiseth 2016, 2017). Genetic relatedness between males and females may affect the resolution of sexual conflict over care. Improved cooperation during care has been suggested to be another fitness‐relevant factor contributing to the evolution of inbreeding tolerance and preferences in biparental species (Thünken et al. 2007a). In accordance with the theoretical predictions (Kokko and Ots 2006), recent studies show that inbreeding is often not avoided (de Boer et al. 2021) but tolerated (Frere et al. 2010) or even preferred (Thünken et al. 2007a; Townsend et al. 2019) in some species (reviewed in Szulkin et al. 2013).

Here, we examine the impact of relatedness between males and females on brood care in the biparental fish *Pelvicachromis taeniatus* , an African cichlid with pronounced inbreeding preferences (Langen et al. 2011; Thünken et al. 2014; Thünken et al. 2007a; Thünken et al. 2007b; Thünken et al. 2011; Thünken et al. 2012; see also Gussone et al. 2023 for similar kin preferences in *Pelvicachromis pulcher* ). The species shows a pronounced social behavior and genetic relatedness between interacting individuals does not only affect mate choice, but also juvenile shoaling and cooperation (Hesse et al. 2015; Hesse and Thünken 2014; Thünken et al. 2020) and intrasexual competition in adults (Gussone et al. 2025; Madge Pimentel et al. 2022; Vitt, Hiller, and Thünken 2020; Vitt, Madge Pimentel, and Thünken 2020). *P. taeniatus* shows mutual mate choice (Thünken et al. 2007a) and after spawning in a cave both sexes contribute to brood care (Thünken et al. 2010). Biparental brood care is a complex behavior in *P. taeniatus* , which includes the selection of a spawning site with suitable natural cavities, modification of the spawning site, defense of the territory, egg care, guidance of the juveniles, and warning the offspring in case of danger (Thünken et al. 2010). During egg care, there is a strong sex‐specific task differentiation. The direct egg care in the cave includes cleaning the eggs or removing dead eggs and is almost exclusively provided by the female. During that period, males guard the cave and fan fresh water into the cave. About 1 week after spawning, the free‐swimming fry leave the cave and are then intensively protected and guided by both parents when swimming as a dense shoal through open water (Thünken et al. 2010). Parents swim in closest proximity to the offspring, which also includes direct physical contact; for example, they shortly take up individual offspring with the mouth or offspring feed on skin mucus of the parents. Furthermore, there is intense communication between parents and the offspring. In case of danger, parents warn the offspring by twitching movements, which causes the immediate sinking to the bottom. Brood care requires fine‐tuned coordination between the parents when alternating in care. As in other cichlids, the reproductive costs are higher for females than for males, indicating an asymmetric outcome of sexual conflict over care (Baldauf et al. 2010, 2011; Goodwin et al. 1998). A previous study reports that kin pairs spent more time protecting the young and increased male cooperation when the female was related (Thünken et al. 2007a). Accordingly, better cooperation in brood care by related parents may render inbreeding an adaptive strategy in this species.

The aim of the present study was to compare male and female contributions to biparental care by related and unrelated pairs. Because parents adjust care to brood quality (Thünken et al. 2010), we used a cross‐fostering approach (Mateo and Johnston 2000), i.e., the exchange of offspring between breeding pairs, to control for potential differences in the performance of inbred and outbred offspring but also for differences in genetic relatedness between parents and offspring (when the parents are related, parents–offspring relatedness is also increased). We randomly assigned inbred and outbred broods to the pairs thereby breaking the link between offspring's inbreeding status and parental relatedness. Assuming better cooperation within related breeding pairs based on kin selection, we predict a more equal contribution of the sexes when parents are related and that related parents spent more time caring than unrelated ones. We additionally conducted an experiment without manipulation of the brood to control for potential effects caused by the cross‐fostering procedure.

## 2 Material and Methods

### 2.1 Experiment 1: Brood Manipulation (Cross‐Fostering)

The animals used in the cross‐fostering experiment were F2‐generation laboratory‐bred fish, originating from wild‐caught animals from the Moliwe River (West Africa, 04° 04′ N/09° 16′ E) in Cameroon (for details concerning breeding and husbandry, see Vitt, Hiller, and Thünken 2020). Before the experiment, individuals were kept in mixed‐sex sibling group tanks (50 cm × 30 cm × 30 cm, length × width × height, l × w × h). Sibling groups had been split into two groups at the age of 1 month and kept separately in two tanks in order to create unfamiliar kin. This was done to be able to examine effects of relatedness independent of familiarity effects. Experimental parental fish were approximately 2 years old when tested. Tanks were separated by opaque, gray plastic sheets and each tank was equipped with a layer of sand as substrate and a water filter (model: Gully from Hobby). The water temperature was kept constant at 25°C ± 1°C. Tanks were illuminated with fluorescent tubes and the light–dark cycle was set to 12:12 h light: dark. Fish were fed with defrosted *Chironomus* larvae and *Artemia* six times a week.

### 2.2 Experimental Set‐Up and Procedure

Kin (i.e., full siblings) or nonkin breeding pairs were arranged and placed each in 50‐l tanks (50 cm × 30 cm × 30 cm, l × w × h), equipped with sand as substrate, a filter (model: Gully, Hobby), a heater (50 W, Hunter) and a ceramic breeding cave (KEROLA). Tanks were visually separated by opaque plastic sheets. Water temperature and illumination conditions were identical to those in the group tanks. Pairs were fed *ad libitum* 6 days a week with defrosted *Chironomus* larvae and *Artemia*.

When two or more pairs had spawned at a similar time point (optimally within 1 week) the parents and the offspring were used for the experiment. Although we arranged 37 breeding pairs, only 15 pairs met these criteria due to high mating failure. Cross‐fostering took place after the parents had cared for their own brood for approx. 2 weeks (i.e., the average age of the broods was 13.8 ± SD 3.1 days). Fifteen pairs received foster offspring; however, two pairs cannibalized the offspring before the start of the experiment. Therefore, the 13 pairs (8 kin and 5 nonkin pairs) that had accepted the foster offspring were used in the experiment. Male body size (75.307 ± 2.750 mm, mean ± SD) and female body size (49.230 ± 2.166 mm, mean ± SD) did not significantly differ between kin and nonkin pairs (males: unpaired *t*‐test, df = 9.982, *t* = 1.543, *p* = 0.154; females: unpaired *t*‐tests, df = 10.643, *t* = 0.042, *p* = 0.967); also, within‐pair size difference did not differ between kin and non‐kin (Wilcoxon test, W = 22, *p* = 0.823). Kin and nonkin pairs did not differ in initial brood size before cross‐fostering (kin: 31.000 ± 2.511; nonkin: 30.625 ± 25.166; unpaired Wilcoxon test, W = 21.5, *p* = 0.883).

For cross fostering, we carefully removed all offspring from a donor breeding pair. The cross‐foster offspring were then randomly assigned to a kin and a nonkin receiver pair whose own offspring had been completely removed directly before. Therefore, cross‐foster parents only cared for the cross‐foster offspring during the experiment. Each cross‐foster offspring group consisted of full siblings only. Cross‐foster parents were unrelated to the cross‐foster offspring; only in one related pair and one unrelated pair was the female an aunt of the cross‐foster brood and in one related pair was the male an uncle of the cross‐foster brood. The broods of the cross‐foster parents were also used for cross fostering. In total, eight different broods were used for cross fostering (six outbred and two inbred broods). Five of these broods provided two cross‐fostering offspring groups each. This resulted in nine outbred and four inbred cross‐fostering groups. We assigned juveniles from five different broods to related pairs and also offspring from five different broods to the unrelated pairs. The eight related pairs received in total five outbred and three inbred cross‐foster offspring groups; the five unrelated pairs received in total four outbred and one inbred cross‐foster offspring group. Cross‐fostered offspring were on average 15.5 ± SD 6.4 days old. Cross‐foster groups consisted of 14–15 individuals, except for one group that consisted of nine juveniles only. Very small groups consisting only of a few offspring are often cannibalized by the parents; however, parents usually cared for offspring with group sizes chosen in this experiment, although they are at the lower end of the natural range.

On the first day of the experimental phase, juveniles were exchanged for cross‐fostering. In detail, the filter and the cave were removed from the tank and all juveniles were carefully caught with a fine‐meshed dip net. Because the parents keep their offspring together by a physical form of calling behavior, that is, head shaking, mostly the whole group could be caught at once. The offspring were transferred to a 1 L plastic box that was filled with aged tap water to a height of about 1.5 cm and the cross‐foster groups were created (see above). Starting 1 day after the cross‐fostering procedure, the parental brood care behavior was examined over four consecutive days. Pairs were video recorded using a Raspberry Pi (Raspberry Pi, Model B+ with a camera module, Raspberry Pi Camera Module v2) for 20 min. Observation was made directly after feeding. A quarter of a self‐adhesive food tablet (JBL Novo Tab, Complete food for carnivorous aquarium fish) was carefully attached centered at the front panel of the tank, approximately 1 cm above the substrate surface. The tablet served as food for the offspring and parents. However, the tablets were not consumed during the observation time. Parents were additionally fed defrosted *Chironomus* larvae and *Artemia* after the trials.

The brood care behavior of 13 pairs was analyzed at four observation days, that is, four data points of male and four4 data points of female brood care for each pair. Videos were analyzed blindly with respect to the treatments. The observer measured the time the female and the male spent guarding the offspring. We considered a parent guarding the offspring when it was in close physical proximity to the offspring, that is, one body length or less away from the offspring (see Thünken et al. 2007a). We used 1 body length as a criterion for guarding behavior to account for the larger males being less moveable compared with the lively females. This guarding behavior is the most reliable measure of brood care at this offspring stage, that is, at the free‐swimming stage, because it occurs, in contrast to other behavior like giving warning signals, with high frequency. During guarding, the parents are constantly paying attention to the offspring: They guide or react to them and stay close to keep the shoal together. Although the exact costs of such guarding behavior are unknown, the high level of parental vigilance will increase stress levels. Additionally, the amount of time spent on guarding behavior comes at the cost of other behaviors, such as foraging. Therefore, we consider offspring guarding as energetically demanding and highly relevant parental brood care behavior.

### 2.3 Experiment 2: Without Brood Manipulation

For this experiment, brood care behavior of related and unrelated pairs was examined without any experimental manipulation (no cross‐fostering, no brood size adjustment). Five related pairs consisting of unfamiliar, full‐siblings, and four unrelated pairs consisting of unfamiliar, unrelated individuals of F4‐generation laboratory‐bred fish were tested. Experimental parental fish were approximately four years old. For four pairs, brood care for two consecutive broods (three related pairs and one unrelated pair) was investigated. Thus, in total thirteen different clutches were used. Pairs were kept as described above in the cross‐fostering experiment. Males and females of related pairs (males: 85.4 ± 4.15 mm; females: 58.4 ± 1.81 mm, mean ± SD) and of unrelated pairs (males: 88.5 ± 1.91 mm; females: 56.5 ± 0.57 mm, mean ± SD) did not significantly differ in size (males: unpaired *t*‐test, *t* = −1.4818, df = 5.853, *p* = 0.190; females unpaired Wilcoxon Test, W = 16, *p* = 0.161); however, there was a slight difference in within‐pair size difference (unpaired *t*‐test, *t* = −2.673, df = 5.25, *p* = 0.042). As soon as the fry were free‐swimming, the brood guarding behavior (see definition in experiment 1) of males and females was directly recorded for 5 days a week for 3 weeks (i.e., fifteen observation days). On observation days, each pair was observed for 5 min. In 20‐s intervals brood care (yes/no) of males and females was recorded. The observer (RA) was unaware of the treatment groups. The fish were fed after the daily observations in the afternoon. Juveniles were fed living *Artemia* larvae and parents defrosted *Chironomus* larvae and *Artemia*.

### 2.4 Data and Statistical Analysis

Statistical analyses were conducted in R (version 4.2.3, R Core Team 2023). Linear mixed‐effects models (LME) were run using the lmerTest package (Kuznetsova et al. 2017). Data for the sex‐specific analysis were arcsin‐transformed to improve normality. For all models, residuals did not significantly deviate from normality.

For the cross‐fostering experiment, data from three observational days were missing because the offspring were cannibalized by the parents (data for male and female care are missing for observation Days 3 and 4 for related pairs and observation Day 4 for an unrelated pair). For the cross‐fostering experiment, for each observation day the proportion of time females, males and both parents spent guarding the offspring was calculated (relative to the observation time of 20 min). For the second experiment without brood manipulation, for each observation day, the proportion of events females, males and both parents guarded the offspring was calculated (relative to the 16 scan points during the daily 5‐min observation). Besides these absolute times, we also calculated the relative times females and males spent guarding the offspring, that is, relative to the total brood care provided by both parents. For both experiments, the absolute and relative brood care of males and females as well as biparental care was averaged across the experimental days.

To examine the individual contributions of males and females in kin and nonkin pairs, LMEs were conducted with the proportion of time an individual spent providing care during the observation as the dependent variable and genetic relatedness (kin/nonkin) and sex (female/male) as fixed factors. Pair ID was entered as a random factor. We entered the interaction between sex and relatedness to examine whether male and female contributions differed between kin and nonkin pairs. Post hoc comparisons were analyzed with the lsmeans package with Tukey *p* value adjustment (Lenth 2016). Furthermore, we compared the amount of biparental care (i.e., when both parents simultaneously provided care) between related and unrelated pairs by applying a linear model (LM, cross‐fostering experiment) and a LME (experiment without manipulation) with biparental care as the dependent variable and relatedness as fixed factors. In the experiment without manipulation, we entered pair ID as a random factor because four pairs were tested with two broods each.

Generally, nonsignificant explanatory variables were stepwise removed from the LMEs in the order of their statistical relevance. Reported *p* values refer to the increase in deviance when the respective variable was removed. Tests of statistical significance were based on likelihood ratio tests (LRT) which follow a *χ* ^2^ distribution. These routines use maximum likelihood parameter estimation.

## 3 Results

**FIGURE 1:**
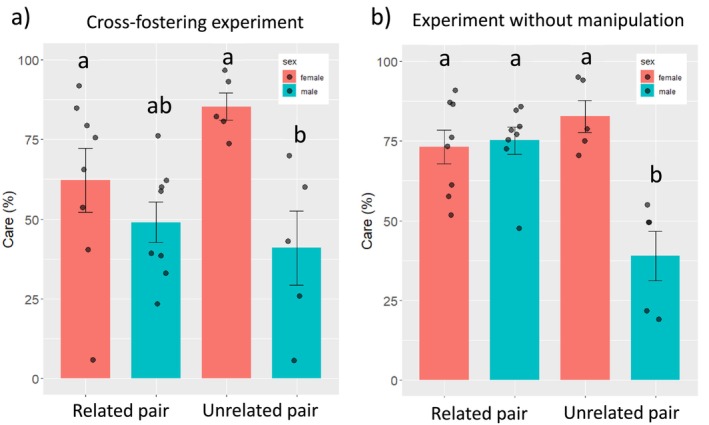
Sex‐specific care and within‐pair relatedness. Absolute time females (red boxes) and males (turquoise boxes) of unrelated and related pairs cared for the offspring in the cross‐fostering experiment (a) and in the experiment without brood manipulation (b). Shown is percentage of time relative to the observation time. Different letters indicate statistically significant differences (*p* < 0.05). Mean values ± SE and individual data are shown.

In both experiments, there was a significant interactive effect between genetic relatedness and sex on individual absolute care (genetic relatedness × sex; Tables 1 and 2, Figure 1). Generally, females cared more than males, when the parents were unrelated, whereas in sibling pairs males and females did not significantly differ in this respect (Tables 3 and 4, Figure 1). In both experiments, female care in related pairs did not significantly differ from female care in unrelated pairs (Tables 3 and 4, Figure 1). Related males cared more than unrelated males in the experiment without brood manipulation (Table 4, Figure 1b), but not in the cross‐fostering experiment (Table 2, Figure 1a). Females in unrelated pairs cared more than males in related pairs in the cross‐fostering experiment but not significantly in the experiment without brood manipulation (Tables 3 and 4, Figure 1). In the cross‐fostering experiment, related females did not significantly differ from unrelated males, whereas in the experiment without manipulation related females provided more care than unrelated males (Tables 3 and 4, Figure 1). Although in both experiments the degree of genetic relatedness did not significantly affect overall parental brood care (Figure 1, Tables 1 and 2), in the control experiment related pairs also showed biparental care more often than unrelated pairs (LME, *χ* ^2^ = 5.067, df = 1, *p* = 0.024); this effect was not found in the cross‐fostering experiment (LM, df = 1, *F* = 0.095, *p* = 0.763).

**TABLE 1:** Cross‐fostering experiment.

Dependent variable	Explanatory variable	Δ DF	*χ* ^2^	*p*
Individual care	**Relatedness × Sex**	**1**	**3.878**	**0.049**
	Relatedness	1	1.218	0.269
	**Sex**	**1**	**8.566**	**0.003**

*Note:* Results of linear mixed effect models examining the impact of parental genetic relatedness on sex‐specific parental care. Random factor: Pair ID. Significant effects are in bold font.

**TABLE 2:** Experiment without brood manipulation.

Dependent variable	Explanatory variable	Δ DF	*χ* ^2^	*p*
Individual care	**Relatedness × Sex**	**1**	**13.458**	**< 0.001**
	Relatedness	1	2.317	0.127
	**Sex**	**1**	**4.615**	**0.032**

*Note:* Results of linear mixed effect models examining the impact of parental genetic relatedness on sex‐specific parental care. Random factor: Pair ID. Significant effects are in bold font.

**TABLE 3:** Cross‐fostering experiment.

Pairs	Estimate	SE	df	t ratio	*p*
Female related—Male related	0.190	0.139	15.4	1.368	0.536
Female related—Female unrelated	−0.334	0.158	30.7	−2.113	0.171
Female related—Male unrelated	0.276	0.158	30.7	1.748	0.317
**Male related—Female unrelated**	**−0.524**	**0.158**	**30.7**	**−3.313**	**0.012**
Male related—Male unrelated	0.086	0.158	30.7	0.547	0.946
**Female unrel.—Male unrel.**	**0.611**	**0.176**	**15.4**	**3.480**	**0.0154**

*Note:* Results of pairwise comparisons with Tukey adjustment. Significant effects are in bold font.

**TABLE 4:** Experiment without brood experiment.

Pairs	Estimate	SE	df	*t* ratio	*p*
Female related—Male related	−0.018	0.091	18.9	−0.201	0.997
Female related—Female unrelated	0.155	0.121	27.4	1.283	0.581
**Female related—Male unrelated**	**−0.441**	**0.121**	**27.4**	**−3.640**	**0.005**
Male related—Female unrelated	0.137	0.121	27.4	1.131	0.673
**Male related—Male unrelated**	**−0.460**	**0.121**	**27.4**	**−3.792**	**0.004**
**Female unrel.—Male unrel.**	**0.597**	**0.116**	**18.9**	**5.152**	**< 0.001**

*Note:* Results of pairwise comparisons with Tukey adjustment. Significant effects are in bold font.

Male and female brood care relative to the total care by both parents also differed between related and unrelated pairs in both experiments (cross‐fostering experiment: LME, sex × relatedness, df = 1, *χ* ^2^ = 4.503, *p* = 0.034, Figure S1; experiment without brood manipulation: LME, sex × relatedness, df = 1, *χ* ^2^ = 26.774, *p* < 0.001, Figure S2). Males and females contributed equally in related pairs (cross‐fostering experiment: LME, df = 1, *χ* ^2^ = 0.475, *p* = 0.491, Figure S1 experiment without brood manipulation: LME, df = 1, *χ* ^2^ = 0.428, *p* = 0.513, Figure S2) whereas in unrelated pairs females provided more care than males (cross‐fostering experiment: LME, df = 1, *χ* ^2^ = 10.703, *p* = 0.001, Figure S1; experiment without brood manipulation: LME, df = 1, *χ* ^2^ = 15.562, *p* < 0.001, Figure S2).

## 4 Discussion

In the present study, we examined female and male contributions to brood care in the cichlid fish *P. taeniatus* in relation to within‐pair genetic relatedness. We conducted two experiments: one cross‐fostering experiment, in which we exchanged the broods between parents, that is, in this experiment the parents were related (full siblings) or unrelated, but parents were not related to the brood. In a second experiment without brood manipulation, related and unrelated parents cared for their own inbred or outbred brood, respectively.

In both experiments, we found that sex‐specific contributions differed depending on the genetic relatedness of the parents (significant interaction between sex and genetic relatedness). While in pairs consisting of unrelated individuals, females provided more care than males; in pairs consisting of related individuals, males and females did not significantly differ and provided a similar amount of care. A higher female contribution, was also found in a previous study that examined care depending on offspring quality and that used unrelated parents only (Thünken et al. 2010). As we find a consistent difference between related and unrelated pairs with respect to sex‐specific contributions to care, although in both experiments the sample size was relatively small, our findings point to a strong effect. Because we controlled for brood effects in the cross‐fostering experiment, the observed effect is most likely caused by the relatedness of the parents. Equal contributions to care from both sexes, could result from females reducing care, males increasing care or a combination of both effects. Post hoc comparisons revealed that in both experiments the female contribution was similar irrespective of whether the male mating partner was related or unrelated. In the cross‐fostering experiment, we did not find significant differences in care between males in related pairs and males in unrelated pairs suggesting that more equal contribution in related pairs resulted from a combination of the effects of reduced female care and increased male care. However, in the experiment with unmanipulated broods, males in related pairs cared more than males in unrelated pairs which could be due to paternal adjustment to female relatedness or to offspring's degrees of relatedness or inbreeding status. This means that based on the present study we cannot disentangle which factors lead to equal parental contributions of males and females in related pairs.

Nevertheless, by demonstrating a balanced contribution of females and males in related pairs, the present study reveals a new aspect of the impact of parental relatedness on brood care in *P. taeniatus* . This study therefore confirms the importance of parental genetic relatedness on brood care in this species. In a previous study it was shown that especially the male contribution was increased, that is, males spent more time guarding the cave where the female cared for the eggs compared to unrelated males, and they also showed less aggression against the partner when she was related (Thünken et al. 2007a). Accordingly, female *P. taeniatus* prefer brothers over unrelated males during mate choice even when he is of poorer phenotypic quality (Thünken et al. 2012). We suppose that females will benefit from cooperative males because it allows the female to recover faster from energetically highly demanding reproduction and, thus, these females may start the next reproductive event earlier than females with less male support. Through the support of related females, males could increase their indirect fitness. More equal contributions of the parents, therefore, might point to better coordination and unity between the female and the male, which could result in more effective brood care and greater safety for the offspring. Even in the absence of predators, differences in brood care intensity might affect offspring performance. For instance, in the closely related *Pelvicachromis pulcher* parental deprivation has been shown to affect shoaling behavior of the offspring (Zacke and Thünken 2024).

In biparental cichlids, male mate desertion has been frequently reported (Jennions and Polakow 2001) because males are less affected by reproduction and thus may abandon the brood earlier than females and could mate with different females. It would be interesting to study whether relatedness affects males' decision to abandon the family. The tighter pair bond in related pairs may result in monogamous relationships that last over several breeding events. Familiarity with the mating partner may further reinforce a pair's coordination and synchronicity in subsequent mating events (Guillette et al. 2016). The absence of extra‐pair paternities in *P. taeniatus* (Langen et al. 2013) may further reduce sexual conflict over reproduction and stabilize the pair bond.

In the previous study, related parents provided more care than unrelated ones (Thünken et al. 2007a). This corresponds to the findings of the control experiment, in which related pairs provided more biparental and overall care (although the latter was statistically not significant) than unrelated pairs. In contrast, in the cross‐fostering experiment we did not find increased care in related pairs. These differences in the results might be explained by a much lower number of observation days in the cross‐fostering experiment compared with the second experiment of the present study and Thünken et al. (2007a), but also by differences in the experimental designs. While cross‐fostering is an elegant method to control for offspring effects on care, the cross‐fostering procedure itself might have a strong impact on parental behavior. Furthermore, fish of the cross‐fostering experiment were fed directly before the observations while in the experiment without brood manipulation and also in Thünken et al. (2007a) fish were fed after the observations. Finally, in Thünken et al. (2007a) and in the experiment without brood manipulation of the present study parental care was observed directly after the offspring were free‐swimming. In contrast, in the cross‐fostering experiment the observations started only about 2 weeks later.

Our results differ from the findings in communal breeding mice (Green et al. 2023), cooperatively breeding cichlids (Zöttl et al. 2013), zebra finch groups (Mathot and Giraldeau 2010), and intraspecific brood parasitism in waterfowl (Andersson et al. 2019), where the inequality of helping increased with relatedness. By compensating for the underinvestment of relatives, individuals can increase their indirect fitness when the relative uses the resources and increase fitness accordingly (Kokko et al. 2002). However, in biparental species inclusive fitness effects may be different than in cooperative breeders. In other biparental species, experimental studies on the impact of relatedness on brood care are missing. The results from observational studies are mixed. Stiver et al. (2008) did not find a significant correlation between within‐pair genetic relatedness and parental effort in a natural colony of a cooperative breeding cichlid. In contrast, Gow et al. (2019) found a positive relationship between the kinship coefficient of a pair and paternal but not maternal effort in song sparrows, which is in accordance with the findings of the present study.

In conclusion, our study showed that relatedness within pairs affects brood care behavior of males and females in a cichlid fish and that genetic relatedness promotes equal contributions of males and females to care. This suggests that genetic relatedness may contribute to solving the sexual conflict over care. Our findings may generally advance our understanding concerning kin‐selected benefits resulting from mating between relatives. Equal contributions of males and females may result in better brood care and ultimately support the evolution of inbreeding preference found in *P. taeniatus* . Generally, the evolution of inbreeding preferences depends on the fitness cost/benefit ratio of mating with kin. Because the costs of inbreeding, that is, inbreeding depression often exceed the benefits, inbreeding is actively avoided (Pike et al. 2021). However, the strength of inbreeding depression can vary. For instance, persistent inbreeding can effectively purge highly deleterious, recessive mutations making inbreeding depression less severe in inbred populations (Hedrick and Garcia‐Dorado 2016). Also, the social environment, such as improved parental care may buffer negative inbreeding effects (Patterson and Pilakouta 2024). Accordingly, we did not find any evidence for strong inbreeding depression so far in *P. taeniatus* (e.g., Thünken et al. 2007a). In contrast, for example, inbred males showed a more intense color ornamentation than outbred ones (Vitt et al. 2022). Still in a competitive environment, inbred males were less able to maintain the intense coloration (Gussone et al. 2025), which may be due to the accumulation of mildly deleterious alleles in inbred individuals. Accordingly, some outbreeding might be advantageous, which might be realized in *P. taeniatus* , for instance, when kin preferences are interacting with preferences for quality traits. We could indeed show that males prefer high‐quality, unrelated females to low‐quality, related females (Thünken et al. 2012) leading to increased genetic diversity in the offspring. Furthermore, kin preference could be weaker in highly inbred individuals, leading to an optimal level of inbreeding as reported in common lizard (Richard et al. 2009). Future studies, will address these issues to further explore the evolution of inbreeding preference.

## Author Contributions

**Timo Thünken:** conceptualization (lead), data curation (equal), formal analysis (equal), funding acquisition (lead), investigation (equal), methodology (equal), project administration (lead), resources (lead), supervision (equal), visualization (lead), writing – original draft (lead), writing – review and editing (lead). **Simon Vitt:** data curation (equal), formal analysis (equal), investigation (equal), methodology (equal), supervision (equal), writing – original draft (supporting). **Janosch Diederich:** data curation (equal), formal analysis (supporting), investigation (equal), methodology (equal). **Ranê Aytaç:** data curation (equal), investigation (equal), methodology (equal).

## Funding

The study was funded by Deutsche Forschungsgemeinschaft grants to T.T. (TH 1615/3–1, TH 1615/3–2, TH 1615/3–3).

## Ethics Statement

The present study adheres to the ASAB/ABS guidelines for the use of animals in research, as well as to the legal requirements of Germany and was conducted in accordance with German laws for animal experiments. Experiments were approved by the regional office for nature, environment and consumer protection North‐Rhine Westphalia (LANUV NRW, reference no. 84e02.04.2015.A580 and 81–02.04.2021.A199).

## Conflicts of Interest

The authors declare no conflicts of interest.

## Supporting information


**Data S1:** Data_brood_careR2.

**Figure S1:** Sex‐specific care and within‐pair relatedness. Proportion of time (relative to pairs total care) females (red boxes) and males (turquoise boxes) of unrelated and related pairs cared for the offspring in the cross‐fostering experiment. Mean values ± SE are shown. Ns indicates *p* > 0.05; **p < 0.01.
**Figure S2:** Sex‐specific care and within‐pair relatedness Proportion of time (relative to pairs total care) females (red boxes) and males (turquoise boxes) of unrelated and related pairs cared for the offspring in the experiment without brood manipulation. Mean values ± SE are shown. Ns indicates *p* > 0.05; ***p < 0.001.

## Data Availability

Data are provided as Supporting Information.
