# Supplementary material for: Genetic Relatedness Promotes Equal Contributions of Males and Females to Brood Care in a Biparental Cichlid Fish
Source: Ecol Evol. 2025 Dec 3;15(12):e72570. doi: 10.1002/ece3.72570 (PMC12675939; doi:10.1002/ece3.72570)
Supplement: Supplementary file 2 — Figure S1: Sex‐specific care and within‐pair relatedness. Proportion of time (relative to pairs total care) females (red boxes) and males (turquoise boxes) of unrelated and related pairs cared for the offspring in the cross‐fostering experiment. Mean values ± SE are shown. Ns indicates p > 0.05; **p < 0.01. Figure S2: Sex‐specific care and within‐pair relatedness Proportion of time (relative to pairs total care) females (red boxes) and males (turquoise boxes) of unrelated and related pairs cared for the offspring in the experiment without brood manipulation. Mean values ± SE are shown. Ns indicates p > 0.05; ***p < 0.001. [file ECE3-15-e72570-s002.docx]

**Supplemental material**

Figure S1

related pairs

unrelated pairs

Proportion of care (%)

0.0

0.2

0.4

0.6

0.8

female

male

ns

**

Cross-fostering experiment

**Fig. S1: Sex-specific care and within-pair relatedness** Proportion of time (relative to pairs total care) females (red boxes) and males (turquoise boxes) of unrelated and related pairs cared for the offspring in the cross-fostering experiment. Mean values ± SE are shown. Ns indicates P > 0.05; ** p <0.01.

related pairs

unrelated pairs

Proportion of care (%)

0.0

0.2

0.4

0.6

0.8

female

male

ns

***

Unmanipulated brood experiment

**Fig. S2: Sex-specific care and within-pair relatedness** Proportion of time (relative to pairs total care) females (red boxes) and males (turquoise boxes) of unrelated and related pairs cared for the offspring in the experiment without brood manipulation. Mean values ± SE are shown. Ns indicates P > 0.05; *** p <0.001.
